# Neurotrophin-3 promotes peripheral nerve regeneration by maintaining a repair state of Schwann cells after chronic denervation via the TrkC/ERK/c-Jun pathway

**DOI:** 10.1186/s12967-023-04609-2

**Published:** 2023-10-17

**Authors:** Xiong Xu, Lili Song, Yueying Li, Jin Guo, Shuo Huang, Shuang Du, Weizhen Li, Rangjuan Cao, Shusen Cui

**Affiliations:** 1 Department of Hand and Foot Surgery, China-Japan Union Hospital of Jilin University, No. 126 Xiantai Street, Changchun, 130033 China; 2 Key Laboratory of Peripheral Nerve Injury and Regeneration of Jilin Province, Changchun, China; 3 Department of Hand & Microsurgery, Peking University Shenzhen Hospital, Shenzhen, China

**Keywords:** Neurotrophin-3, c-Jun, Chronic denervation, Peripheral nerve regeneration, Repair Schwann cell, ERK pathway

## Abstract

**Background:**

Maintaining the repair phenotype of denervated Schwann cells in the injured distal nerve is crucial for promoting peripheral nerve regeneration. However, when chronically denervated, the capacity of Schwann cells to support repair and regeneration deteriorates, leading to peripheral nerve regeneration and poor functional recovery. Herein, we investigated whether neurotrophin-3 (NT-3) could sustain the reparative phenotype of Schwann cells and promote peripheral nerve regeneration after chronic denervation and aimed to uncover its potential molecular mechanisms.

**Methods:**

Western blot was employed to investigate the relationship between the expression of c-Jun and the reparative phenotype of Schwann cells. The inducible expression of c-Jun by NT-3 was examined both in vitro and in vivo with western blot and immunofluorescence staining. A chronic denervation model was established to study the role of NT-3 in peripheral nerve regeneration. The number of regenerated distal axons, myelination of regenerated axons, reinnervation of neuromuscular junctions, and muscle fiber diameters of target muscles were used to evaluate peripheral nerve regeneration by immunofluorescence staining, transmission electron microscopy (TEM), and hematoxylin and eosin (H&E) staining. Adeno-associated virus (AAV) 2/9 carrying shRNA, small molecule inhibitors, and siRNA were employed to investigate whether NT-3 could signal through the TrkC/ERK pathway to maintain c-Jun expression and promote peripheral nerve regeneration after chronic denervation.

**Results:**

After peripheral nerve injury, c-Jun expression progressively increased until week 5 and then began to decrease in the distal nerve following denervation. NT-3 upregulated the expression of c-Jun in denervated Schwann cells, both in vitro and in vivo. NT-3 promoted peripheral nerve regeneration after chronic denervation, mainly by upregulating or maintaining a high level of c-Jun rather than NT-3 itself. The TrkC receptor was consistently presented on denervated Schwann cells and served as NT-3 receptors following chronic denervation. NT-3 mainly upregulated c-Jun through the TrkC/ERK pathway.

**Conclusion:**

NT-3 promotes peripheral nerve regeneration by maintaining the repair phenotype of Schwann cells after chronic denervation via the TrkC/ERK/c-Jun pathway. It provides a potential target for the clinical treatment of peripheral nerve injury after chronic denervation.

**Supplementary Information:**

The online version contains supplementary material available at 10.1186/s12967-023-04609-2.

## Introduction

Peripheral nerve injury is a common clinical condition and an important factor leading to serious disability and economic loss. Although peripheral nerves can regenerate after injury, nerve regeneration in the clinic is suboptimal, making it an urgent problem that must be addressed in clinical practice [1, 2].

Following peripheral nerve injury, Wallerian degeneration occurs at the injured distal stump and the axons disintegrate. Myelin and Remak Schwann cells transform into Schwann cells with the repair phenotype [3]. These repair Schwann cells clear myelin through autophagy. They secrete nerve growth factors and form regeneration tracks (called bands of Büngner) which promote and guide axonal growth from the proximal end of the injury to the distal end and re-innervate target organs [2]. During this process, the repair Schwann cells provide an essential foundation and environment for the survival of neurons and axon regeneration [3–6]. However, if no axons grow into the bands of Büngner distal to the injury site over an extended time, the Schwann cells lose the repair ability.

In many rodent models, the duration of denervation is short because of the relatively close distance between the injury site and innervated target organ. Axons re-innervate target organs in a relatively short period through the bands of Büngner formed by repair Schwann cells. However, for human peripheral nerve injury, owing to the relatively long distance between the injured site and the target organ and the limited regenerative capacity of axons, it takes a long time to re-innervate the target organ, which results in chronic denervation and poor nerve regeneration. Therefore, maintaining the repair phenotype of repair Schwann cells during chronic denervation has become a therapeutic target for peripheral nerve injury.

c-Jun, a component of the transcription factor activator protein-1, plays an important role in maintaining the repair phenotype of repair Schwann cells [7]. Removal of c-Jun from denervated Schwann cells impairs the repair function and inhibits axonal regeneration [7, 8], whereas, stable overexpression of c-Jun in Schwann cells promotes nerve regeneration [9–11]. However, c-Jun acts as a negative regulator of myelination [10, 12, 13] and sustained overexpression carries a risk of inhibiting remyelination. Also, high expression of c-Jun has been observed in schwannomas [14]. Overall, overexpression of c-Jun in Schwann cells is not practical for the treatment of peripheral nerve injury after chronic denervation. Therefore, identifying the molecular signaling pathway that upregulates c-Jun may provide a potential therapeutic target for treating peripheral nerve injury.

Previous studies have shown that c-Jun in Schwann cells is mainly upregulated by p38 mitogen-activated protein kinase (p38 MAPK), extracellular signal-regulated kinase (ERK), and Jun N-terminal kinase (JNK) signaling pathways [15–19], and NT-3, a neurotrophic factor, has the potential to activate aforementioned signaling pathways [20–23]. NT-3 can be transported from the muscle to the central nervous system (CNS) and peripheral nervous system (PNS) through systemic circulation [24–26] and has good clinical application prospects. Peripheral therapy with recombinant NT-3 has been tested in phase I and II clinical trials and was found to be safe and well-tolerated [27–29]. However, whether NT-3 can upregulate c-Jun to promote peripheral nerve regeneration after chronic denervation, especially when c-Jun is rarely expressed in the injured distal nerve, and the potential mechanisms of action are largely unknown.

Here, we found that c-Jun was first upregulated and maintained at a high level until 5 weeks after denervation in the distal stumps, and decreased thereafter. NT-3 maintained the high expression of c-Jun if given at the 5th week, which promoted the regeneration of axons after 10-week denervation. Silencing of c-Jun impaired the promotion effect of NT-3. More importantly, c-Jun was upregulated again after 8 weeks of denervation when the expression of c-Jun was very low. Additionally, increased c-Jun also promoted axon regeneration in the chronic denervation model (10-week denervation). Further studies found that the administration of NT-3 activated the ERK pathway, which mediates the high expression of c-Jun in Schwann cells. TrkC has a high affinity for NT-3 and has been demonstrated to be the receptor for NT-3 in the regulation of c-Jun. In summary, this study identified the TrkC/ERK pathway as the major molecular mechanism for NT-3 to upregulate c-Jun and provide a potential therapeutic target for the clinical treatment of peripheral nerve injury after chronic denervation.

## Materials and methods

### Drugs and reagents

Human recombinant NT-3 (#C079) was provided by Novoprotein (Suzhou, China) and stored in powder form at − 80 °C. PD98059 (#T2623) was obtained from TargetMol (MA, USA). K-252a (#05288) and Dimethyl sulfoxide (DMSO) (#D2650) were purchased from Sigma-Aldrich (MO, USA). Details of the sources and uses of the antibodies are provided in Additional file 1: Table S1.

### Animals

Male adult Sprague Dawley rats (220–250 g, 8-week-old) were obtained from the Experimental Animal Center of Jilin University. Food and water were available ad libitum and all rats were raised in an environment with a controlled indoor temperature of 22 °C ± 2 °C, humidity of 50–60%, and 12 h light/dark cycles. All experimental procedures were conducted in strict accordance with the Guide for the Care and Use of Laboratory Animals established by the National Institutes of Health in the United States and approved by the Animal Ethics Committee of the Basic Medical College of Jilin University. Each experimental group consisted of randomly selected samples (n = 3 or 4). Sample selection was based on recognized standards from previous professional knowledge and literature reviews.

### Surgery

All surgical experiments were performed under 2.5% isoflurane anesthesia. To explore the regenerative effects of NT-3 on peripheral nerves after chronic denervation, a chronic denervation regeneration model was established [30] (Additional file 2: Fig. S1a). Firstly, we exposed the sciatic nerve and then separated and freed the common peroneal and tibial nerves from the point of bifurcation of the sciatic nerve. We severed the common peroneal nerve and fixed the distal stump onto nearby muscles, leaving the distal stump in a denervated state. After 5 or 8 weeks, we administered NT-3, NT-3 + AAV, or phosphate buffer saline (PBS) to the distal denervated nerve. Ten weeks after denervation, the scar tissue at the distal stump of the chronic denervated common peroneal nerve was removed, leaving a distance of 0.4 cm from the incision to the lateral margin of the peroneus muscle. The tibial nerve was severed and sutured to the distal stump of the chronic denervated common peroneal nerve, and NT-3 or PBS was continued. Six weeks after suturing, we examined the peripheral nerve regeneration as in the following subsections. We simultaneously severed the common peroneal and tibial nerves and immediately sutured the proximal end of the tibial nerve to the distal end of the common peroneal nerve as the positive control group (Additional file 2: Fig. S1b). NT-3 was administered by intramuscular injection around the common peroneal nerve every other day, and AAV was injected directly into the denervated common peroneal nerve using a microinjection needle (Hamilton, Switzerland).

### Fluorescein isothiocyanate-conjugated Cholera toxin subunit B (FITC-CTB) retrograde tracing

Briefly, FITC-CTB retrograde tracing was conducted according to a previously described protocol [31, 32] by injecting 10 µl of 0.4 mg/ml FITC-CTB (Absin, Shanghai, China) into two sites of the anterior tibialis muscle on the injured side of rats under anesthesia. After 7 days, the rats were perfused with 0.01 M PBS followed by 4% paraformaldehyde (PFA). The lumbar (L) spinal segments (L4 and L5) and corresponding dorsal root ganglion (DRG) were fixed in 4% PFA at 4 °C for 24 h. The fixed tissue was incubated overnight in 30% sucrose and embedded in OCT compound. The samples were sectioned transversely at 12 μm thicknesses using a freezing microtome (Leica, Wetzlar, Germany). The samples were immunofluorescence stained with rabbit polyclonal anti-NeuN antibody and treated with secondary antibodies conjugated with Alexa Fluor® 546. The stained samples were observed under a laser-scanning confocal microscope (Nikon, Osaka, Japan). The percentage of FITC-CTB-labeled motor neurons in the spinal ventral horn and sensory neurons in the DRG was calculated.

### Determination of the muscle wet weight ratio and analysis of neuromuscular junction (NMJ) reinnervation

Briefly, after anesthetizing the rats, the anterior tibial muscles on the injured and uninjured sides were weighed to calculate the wet weight ratio (wet weight of injured muscle/wet weight of uninjured muscle). The injured anterior tibial muscles were fixed with 4% PFA for 24 h and the diameter of the muscle fibers was analyzed by H&E staining. The rats were then perfused with 0.01 M PBS followed by 4% PFA, and the extensor digitorum longus was fixed in 4% PFA for 24 h. The muscle was divided into small bundles using a stereomicroscope. The samples were washed four times with 0.01 M PBS, at 10 min intervals, treated with 0.1 M glycine for 30 min and washed four more times with 0.01 M PBS at 10 min intervals. They were permeabilized with 2% Triton-100/PBS for 48 h, blocked with 5% Bovine Serum Albumin (BSA) + 2% Triton-100 in PBS for 30 min, and then incubated with primary anti-neurofilament (NF) and synapsin (Syn) antibodies overnight at 4 °C. The samples were washed and incubated with the appropriate secondary antibodies (goat anti-rabbit IgG, Alexa Fluor 488)or Fluor 594-conjugated α-bungarotoxin (α-BTX) for 1 h at room temperature. Images were acquired using a laser-scanning confocal microscope (Nikon, Tokyo, Japan).

### Schwann cell cultures

Rat Schwann cells were cultured as described previously [33]. Briefly, sciatic and brachial plexus nerves of 2-day-old rats were minced and digested with collagenase and pancreatin. The cells were then purified and passaged in DMEM supplemented with 10% FBS, 10 ng/ml Neuregulin-1 (Nrg-1), 2 µM forskolin, penicillin (100 IU/ml), and streptomycin (100 IU/ml) on poly-l-lysine (PLL)-coated plates at 37 °C in a humidified incubator with 5% CO_2_. On the day preceding the administration of NT-3 to the passaged Schwann cells, the culture medium was refreshed with a complete medium lacking Nrg-1 and forskolin, in accordance with the previously outlined procedure.

### Western blotting

Samples of peripheral nerves and Schwann cells were prepared using RIPA lysis buffer (#C500008; Sangon Biotech, Shanghai, China). Protein concentrations were determined using the bicinchoninic acid assay (#P0010, Beyotime Biotechnology). Protein samples were separated by 10% SDS-PAGE and transferred onto PVDF membranes. The membranes were blocked with 5% BSA in tris-buffered saline with Tween 20 (TBST) for 1.5 h at room temperature and incubated with primary antibodies overnight at 4 °C. Immune complexes were detected using HRP conjugated to a secondary antibody for 2 h at room temperature. Finally, images were scanned using a GS800 Densitometer Scanner. Blot intensity was assessed using Multi Gauge software (Fuji, Tokyo, Japan). GAPDH (glyceraldehyde 3-phosphate dehydrogenase) and β-tubulin were used as controls to normalize the protein levels.

### Immunofluorescence staining

Immunofluorescence staining of cultured cells was carried out by adding medium containing approximately 30,000 Schwann cells to a small circular glass with a diameter of 1.5 cm coated with PLL. After the cells reached an appropriate density (80%), they were washed twice with 0.01 M PBS and fixed with 4% PFA for 10 min. They were then washed twice in 0.01 M PBS for a total of 10 min. The cells were blocked with 2% BSA + 0.3% Triton X-100 in PBS for 30 min. Nerve samples were fresh-frozen during embedding in OCT and cut into 12 μm sections. After the samples were air-dried, they were washed thrice with PBS for 10 min each and blocked with 5% BSA + 1% Triton X-100 in PBS for 30 min. The samples were then incubated with the primary antibody overnight at 4 °C. The next day, the samples were washed with 0.01 M PBS and incubated with the appropriate secondary antibodies conjugated to fluorescent probes for 1 h at room temperature. The samples were then washed and, if necessary, stained with DAPI (1:1000) to identify cell nuclei. Images were acquired using a laser-scanning confocal microscope (Nikon, Tokyo, Japan).

### Designing small hairpin RNA (shRNA) sequences and constructing AAV vectors

The shRNA sequences were designed to target the expression of c-Jun, and a non-interfering control shRNA was designed. The constructed shRNAs were inserted into the selected interference vector (pAAV-U6-shRNA/spgRNA v2.0-CMV-EGFP-WPRE) (Additional file 2: Fig. S2). Both shRNAs were cloned into the pAAV plasmid under the control of the U6 promoter and expressed as EGFP under the CMV promoter (pAAV-sh1 and pAAV-sh2). A control vector expressing a non-targeting shRNA (pAAV-U6-shCtrl-CMV-eGFP) was used as a control. AAV2/9 (1 × 10^11^ viral genomes (VG)/nerve) was directly injected into the 5-week distal denervated stump of the common peroneal nerve using a microinjector (Hamilton, Switzerland) under 2.5% isoflurane anesthesia. After the administration of NT-3 for 5 weeks, the expression of c-Jun in the distal nerve was evaluated.

The c-Jun shRNA sequences (sense strand) were as follows: sh1, 5′-GCCCTAGCTGAACTGCATA-3′; sh2, 5′-CCAACCTCAGCAACTTCAA-3′; and shCtrl, 5′-CCTAAGGTTAAGTCGCCCTCG-3′. These procedures were performed by OBiO Technology (Shanghai, China).

### Small-interfering RNA (siRNA) transfection

siRNA sequence design and synthesis were provided by GenePharma (Suzhou, China). Two siRNAs targeting tropomyosin receptor kinase C (TrkC) and a non-interfering siRNA were designed. Lipofectamine 3000 transfection reagent (L3000150, Invitrogen) was used to transfect the cultured Schwann cells. The cells were transfected with siRNA for 6 h, and the medium was then replaced. Three days after transfection, cellular proteins were extracted for further analysis. The siRNA sequences were as follows: Si-control, 5′-UUCUUCGAACGUGUCACGUTT-3′; Si-TrkC(1), 5′-GCAUCAACAUCACGGACAUTT-3′; Si-TrkC(2), 5′-GGACCAAUGUACAUGCCAUTT-3′.

### TEM

Small segments of the distal stumps of the regenerated nerves were fixed in 2.5% glutaraldehyde after perfusion. After fixation, the sections were treated with 1% osmium tetroxide solution for 1 h, washed, and dehydrated using graded alcohol solutions. The samples were then embedded in Epon 812 epoxy resin and cut into ultrathin sections of 60 nm. The sections were stained with lead citrate and uranyl acetate before observation under a TEM (JEOL Ltd., Tokyo, Japan). The g-ratio was used to analyze the myelination of regenerated axons.

### Cell viability assay

The Cell Counting Kit-8 (CCK-8) assay was used to evaluate the toxicity of NT-3 treatment at different timepoints. Briefly, Schwann cells were seeded into 96-well plates at a density of 4000 cells/well in 100 µl of the complete medium and cultured at 37 °C in a humidified incubator with 5% CO_2_. After cell adhesion, NT-3 (50 ng/ml) or PBS (vehicle control) was administered to the cultured Schwann cells (eighth passage) for 8, 16, 24, and 48 h. At the end of each experiment, 10 µl CCK-8 reagent (Dojindo, Kyushu Island, Japan) was added into each well. The cells were further cultured and protected from light for 2 h at 37 °C and the optical density value (OD 450nm) was measured using a Multiskan microplate reader (Thermo Fisher Scientific, Waltham, MA, USA).

### Statistical analysis

Analyses were performed by an investigator who was blinded to the treatment groups. Statistical analyses were performed using GraphPad Prism 9.4.1 software. An unpaired two-tailed t-test was used when comparing the mean between two independent groups. Analysis was performed using one-way analysis of variance (ANOVA) followed by Dunnett’s or Tukey’s post hoc tests, as indicated for multiple groups. All data are presented as the mean ± standard error of the mean (SEM). Differences were considered statistically significant when P values were less than 0.05.

## Results

### NT-3 upregulates the expression of c-Jun in Schwann cells in vitro

**Fig. 1:**
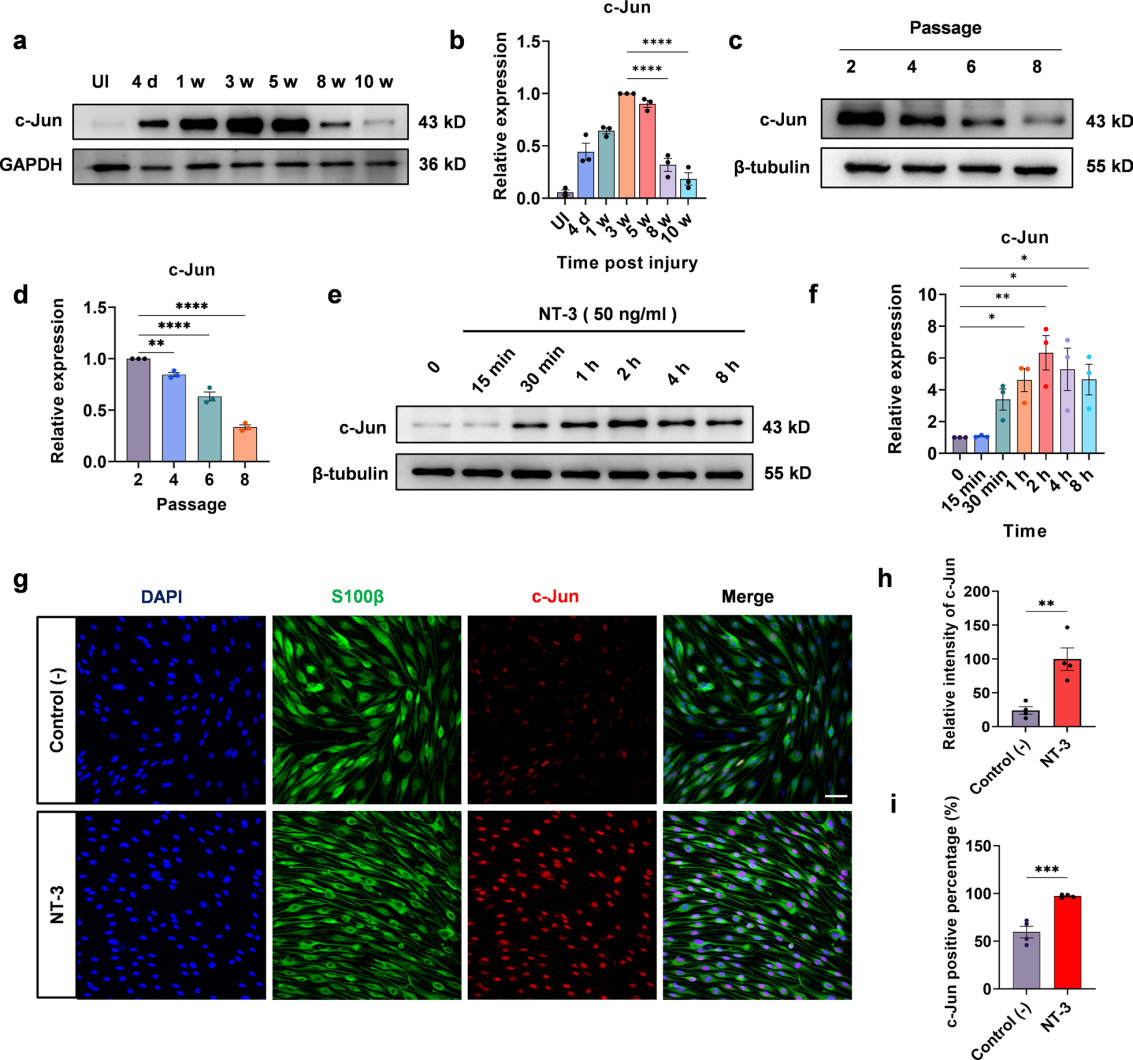
NT-3 upregulates the expression of c-Jun in Schwann cells.** a** Western blotting was used to detect the expression of c-Jun in the uninjured and injured distal stumps after 4 days and at 1, 3, 5, 8, and 10 weeks of denervation. **b** Quantification of the relative expression of c-Jun in **a**. Data were normalized to 3 weeks post-injury. One-way ANOVA with Dunnett’s multiple comparisons test, ****P < 0.0001, n = 3. **c** Western blot of c-Jun in cultured Schwann cells at passages 2, 4, 6, and 8. **d** Quantification of the relative expression of c-Jun in **c**. Data were normalized to passage 2. One-way ANOVA with Dunnett’s multiple comparisons test, **P < 0.005, ****P < 0.0001, n = 3. **e** The expression of c-Jun at different time points after administration of NT-3 analyzed using western blotting. **f** Relative quantitative analysis of c-Jun expression shows that NT-3 could upregulate c-Jun expression. Data normalized to 0 (control group). One-way ANOVA with Dunnett’s multiple comparisons test, *P < 0.05, **P < 0.005, n = 3. **g** Immunofluorescence analysis of c-Jun expression in Schwann cells; scale bar, 50 μm. **h** The expression of c-Jun in Schwann cells analyzed using fluorescence intensity. The expression of c-Jun in the NT-3 group is significantly upregulated compared to that in the control group. Unpaired Student’s t-test, **P < 0.005, n = 4. **i** Percentage of c-Jun positive Schwann cells. Unpaired Student’s t-test, ***P < 0.001, n = 4. All numerical data are presented as mean ± SEM

To determine the relationship between the expression of c-Jun in the injured distal stumps and the time of denervation, the common peroneal nerve was cut, and the distal stump was fixed to the adjacent muscle so that the distal stump was denervated. The expression of c-Jun in the uninjured and injured distal stumps was measured at 4 days, 1, 3, 5, 8, and 10 weeks after the surgery (Fig. 1a, b). Western blotting showed that c-Jun was almost undetectable in uninjured nerves; however, in the distal stumps its expression gradually increased from day 4, peaked at week 3, and maintained a high level at week 5. Subsequently, c-Jun began to decrease and was seldom expressed at 10 weeks (Fig. 1b).

It has previously been shown that changes in c-Jun occur predominantly in repair Schwann cells of the distal denervated nerve stump [9]. To confirm this finding, Schwann cells from the sciatic and brachial plexus nerves of 2-day-old rats were isolated and cultured. Chronic denervation was mimicked by subculturing Schwann cells in vitro. Western blotting showed that c-Jun was abundant at the second passage and then gradually decreased from the fourth passage to the eighth passage (Fig. 1c, d), suggesting that c-Jun decreased in chronically denervated Schwann cells.

Next, we explored whether NT-3 could upregulate c-Jun expression. NT-3 (50 ng/ml) was administered to mimic denervated Schwann cells (eighth passage) and the expression of c-Jun was detected 15 min, 30 min,1 h, 2 h, 4 h, and 8 h later (Fig. 1e). Our results showed that, compared with the control (0 min), c-Jun expression increased 30 min later and was approximately sixfold higher at 2 h (Fig. 1f). The expression of c-Jun in Schwann cells was further detected by immunofluorescence staining with DAPI/c-Jun/s100β at 0 min and 2 h after NT-3 treatment (Fig. 1g), and c-Jun was located in the nuclei of the cultured Schwann cells. The relative fluorescence intensity of c-Jun was approximately fourfold higher at 2 h than that of the control group (0 min) (Fig. 1h). In addition, the number of c-Jun positive Schwann cells was also higher (Fig. 1i). Cell viability was evaluated by CCK-8 and NT-3 showed no toxic effects on Schwann cells (Additional file 2: Fig. S3). Overall, the above results suggest that NT-3 could upregulate the expression of c-Jun in Schwann cells in vitro.

### NT-3 promotes peripheral nerve regeneration after chronic denervation by maintaining high levels of c-Jun in the distal denervated nerves

**Fig. 2:**
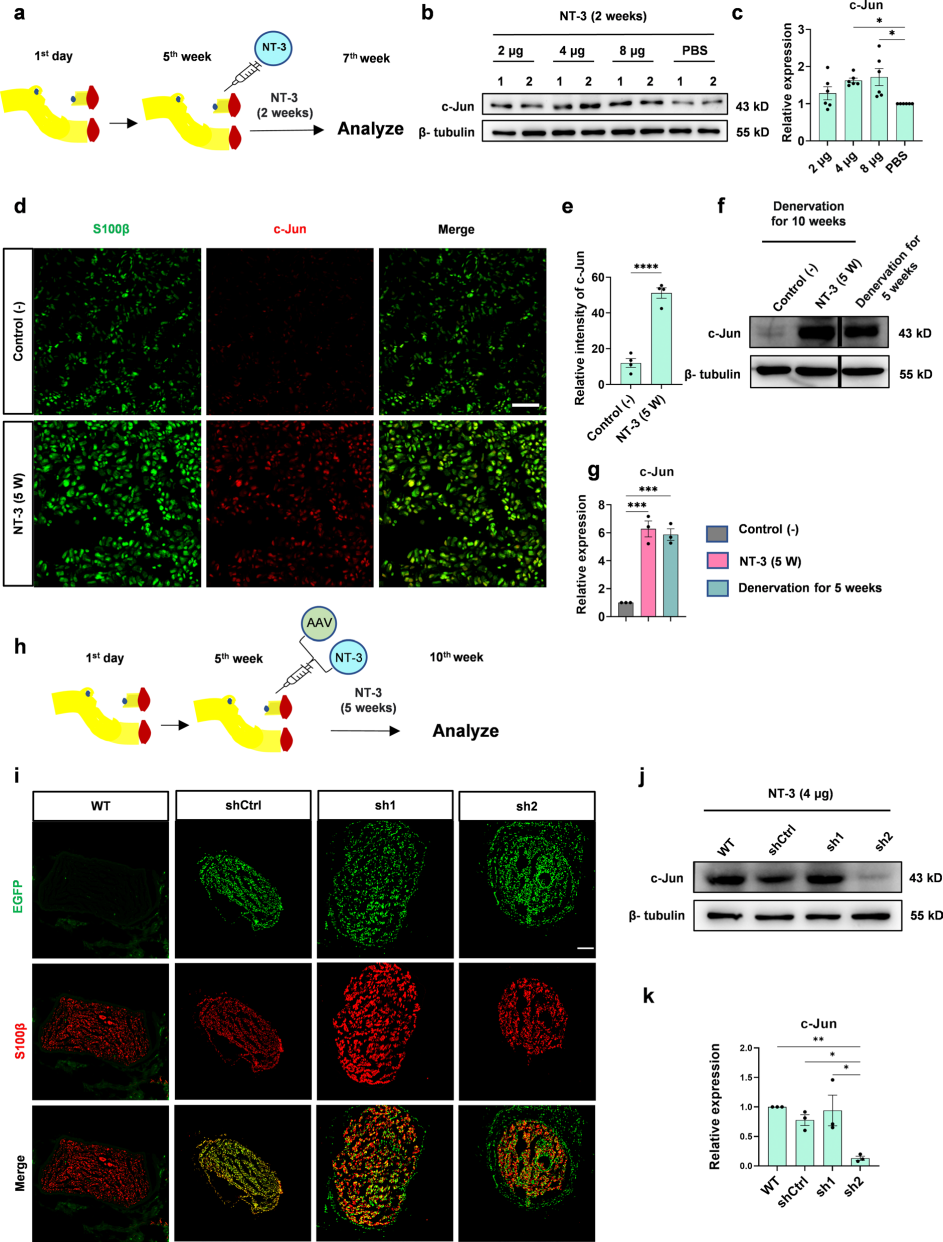
NT-3 maintains the high expression of c-Jun in the denervated distal nerve.** a** Operational diagram of administration of NT-3 at a total of 2, 4, and 8 µg in 50 µl PBS. **b** The expression of c-Jun was analyzed using western blotting. **c** Relative quantitative analysis of the expression of c-Jun. Data normalized to the PBS group. One-way ANOVA with Tukey’s multiple comparison test, *P < 0.05, n = 6. **d** Immunofluorescence analysis of c-Jun expression in the distal denervated nerve. Scale bar, 50 μm. **e** The expression of c-Jun analyzed by fluorescence intensity. The expression of c-Jun in the NT-3 (5 W) group is significantly upregulated compared with that in the control (−) group. Unpaired Student’s t-test, ****P < 0.0001, n = 4. **f** Western blotting used to analyze the expression level of c-Jun in the NT-3 (5 W) and control (−) groups and the distal denervated nerves for 5 weeks. All protein lanes were run on the same gel but were non-contiguous. **g** Relative quantitative analysis of c-Jun expression shows that NT-3 can maintain a high level of c-Jun. Data were normalized to the control (−) group using one-way ANOVA with Tukey’s multiple comparison test, ***P < 0.001, n = 3. **h** Schematic showing the location and time of AAV2/9 and NT-3 administration. **i** Immunofluorescence staining used to analyze the infection efficiency and characteristics of AAV2/9 in the denervated distal stump. Scale bar, 100 μm. **j** Western blotting used to detect the expression of c-Jun in each group. **k** Relative quantitative analysis of c-Jun expression shows that sh2 significantly inhibited the expression of c-Jun. Data normalized to WT, one-way ANOVA with Tukey’s multiple comparison test, *P < 0.05, **P < 0.005, n = 3. All numerical data are presented as mean ± SEM

Because c-Jun began to decrease from 5 weeks after denervation and NT-3 upregulated the expression of c-Jun in cultured Schwann cells, we questioned whether NT-3, if given at this time point, could prevent the decline of c-Jun in vivo. Five weeks after denervation, different doses of NT-3 (in 50 µl PBS) were administered locally every other day by intramuscular injection around the distal stumps for 2 weeks (Fig. 2a). PBS was administered in the control group. Western blotting showed that, compared with the control (PBS), both 4 and 8 µg NT-3 prevented the decline of c-Jun (Fig. 2b, c).

Then, we further questioned whether NT-3 could maintain this high level of c-Jun during chronic denervation (10-week denervation). To address this hypothesis, 4 µg NT-3 (in 50 µl PBS) was administered locally every other day from the 5th week after denervation by intramuscular injection for 5 weeks to the NT-3 (5 W) group. Immunofluorescence staining showed a well-organized cellular morphology in the NT-3 (5 W) group, accompanied by more s100β + cells expressing c-Jun (Fig. 2d). Quantification of the immunofluorescence intensity showed that the expression of c-Jun in the distal stumps was higher in the NT-3 (5 W) group than in the control (−) group (Fig. 2e). Meanwhile, western blotting confirmed the high expression of c-Jun at 5 weeks after denervation, and this high level lasted for 10 weeks following NT-3 treatment (Fig. 2f, g). These results showed that the administration of NT-3 could maintain the high expression of c-Jun and protected it from decreasing during chronic denervation.

Next, to explore whether NT-3 can promote peripheral nerve regeneration by maintaining a high level of c-Jun after chronic denervation, two shRNAs targeting c-Jun (sh1 and sh2) and a control non-interfering shRNA (shCtrl) were constructed and packed into AAV2/9, which has been reported to have high infection efficiency in Schwann cells of rat sciatic nerves [34]. After 5 weeks of denervation, AAV2/9 carrying sh1, sh2, or shCtrl (1 × 10^11^ VG/nerve) was injected into the distal denervated stumps. Simultaneously, 4 µg (in 50 µl PBS) NT-3 was administered every other day for 5 weeks (Fig. 2h). Distal stumps were harvested for analysis, and high infection efficiency was observed by immunofluorescence staining. AAV2/9 mainly infected S100β positive Schwann cells and sh2 induced an obvious morphological change where the distribution of Schwann cells was disorganized (Fig. 2i). The silencing efficiency was tested by western blotting, and sh2 showed a higher silencing rate and was chosen for the following studies (Fig. 2j, k).

**Fig. 3:**
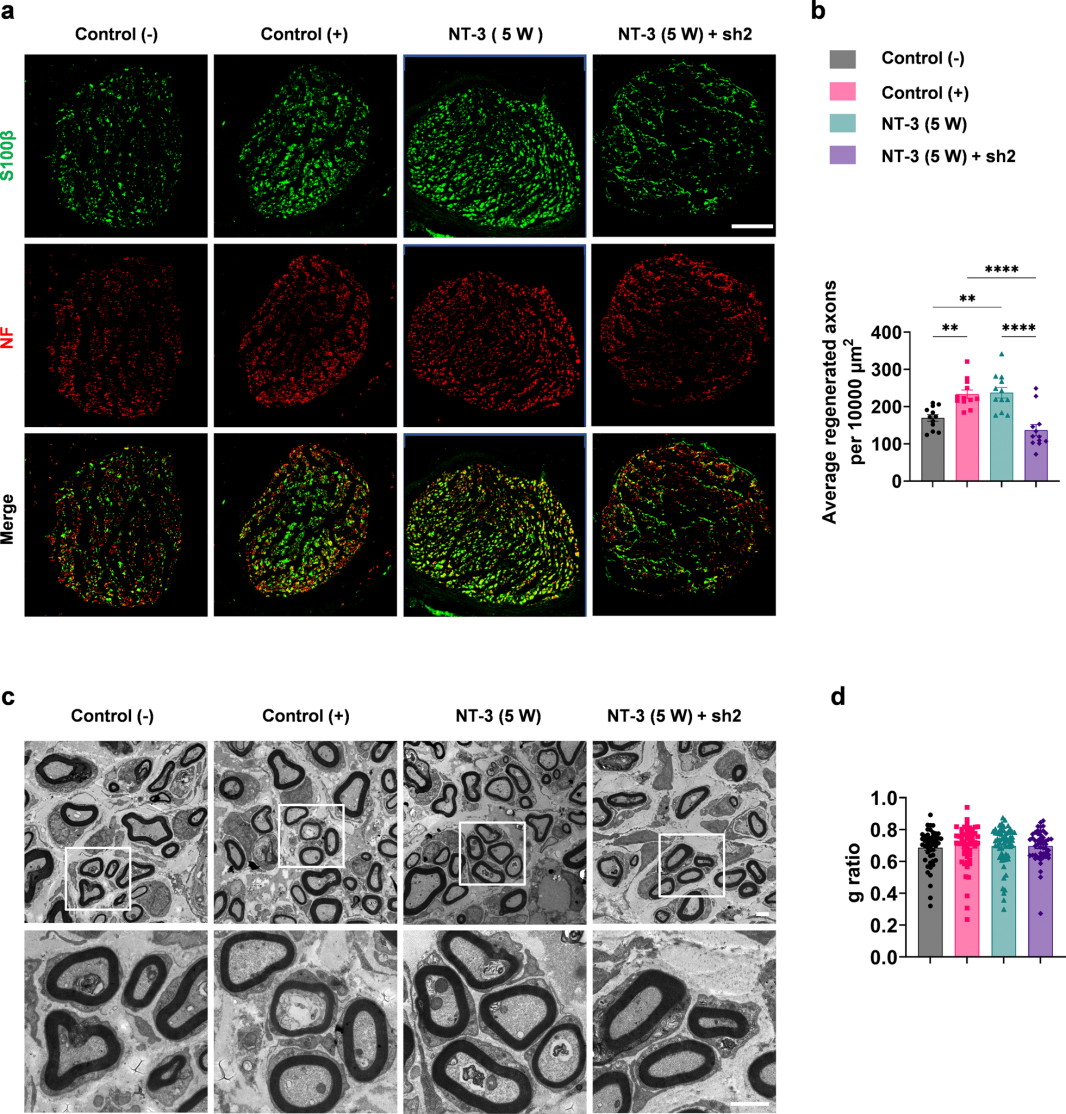
NT-3 promotes peripheral nerve regeneration after chronic denervation by maintaining high levels of c-Jun.** a** Representative images of peripheral nerve regeneration observed using immunofluorescence staining. S100β labeled myelin and NF-labeled axons; scale bar, 100 μm. **b** Number of regenerated axons per 10,000 µm^2^ in each group, n = 12 from 4 rats. **c** Representative images of myelination of regenerated axons in each group observed by TEM; scale bar, 2 μm. **d** Statistical analysis of myelination in each group, n = 48 or 63 from 3 rats. All numerical data were analyzed using one-way ANOVA with Tukey’s multiple comparison test and are presented as mean ± SEM. **P < 0.005, ****P < 0.0001

A peripheral nerve regeneration model was established to investigate whether NT-3 promotes nerve regeneration after chronic denervation (Additional file 2: Fig. S1a) [9, 30]. Briefly, the common peroneal nerve was transected and the injured distal nerve was denervated for 10 weeks. The tibial nerve was then transected, and its proximal stump was sutured to the distal denervated stump of the common peroneal nerve. NT-3 (4 µg in 50 µl PBS) was administered alone or together with c-Jun sh2 at the distal common peroneal stump 5 weeks after denervation. PBS injection served as a negative control (−) group, and rats receiving immediate suture between the proximal stump of the tibial nerve and the distal stump of the common peroneal nerve served as positive controls (Additional file 2: Fig. S1b). Six weeks later, a 2 mm-segment of nerve located 1 cm distal to the suture site was excised and subjected to immunofluorescence staining with anti-NF antibodies to label axons. The number of average regenerated axons per unit area (10,000 µm^2^) was quantified in the NT-3 treated rats, which was similar to that of the positive control. In contrast, silencing of c-Jun by AAV simultaneously with NT-3 administration (NT-3 (5 W) + sh2) reversed the promoted regeneration (Fig. 3a, b). TEM was used to check the myelination of regenerated nerves. However, no obvious difference was observed among these groups by analyzing the g-ratio, suggesting that NT-3 did not affect remyelination (Fig. 3c, d).

**Fig. 4:**
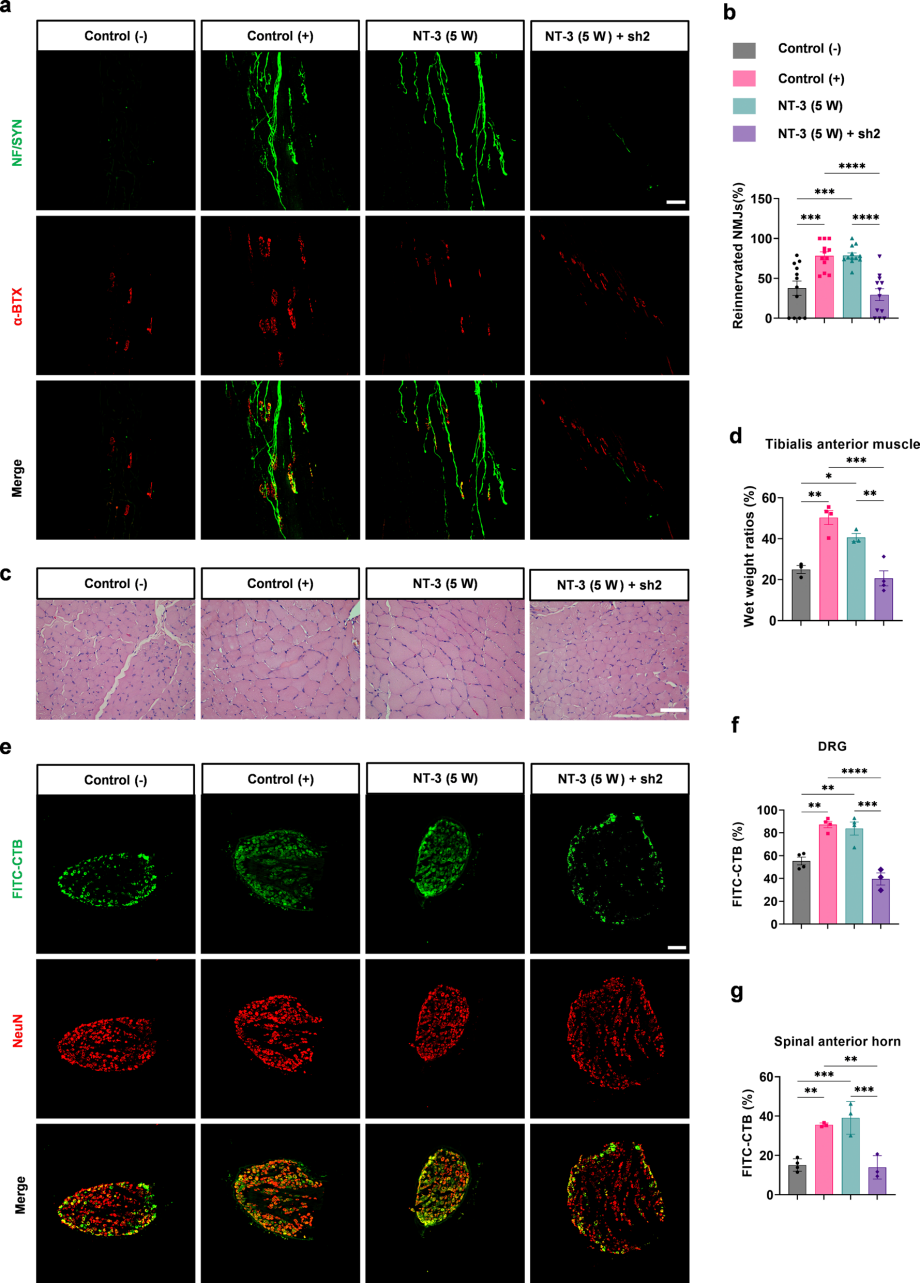
Reinnervation of target muscles by regenerated axons.** a** Representative images of NMJs using immunofluorescence staining. Green: Presynaptic NMJs and regenerated axons. Red: AChRs in the post-synapses of NMJs; scale bar, 100 μm. **b** Percentage of reinnervated NMJs per group, n = 12 from 3 rats. **c** the diameter of the anterior tibial muscle fibers in each group was shown by H&E staining, scale bar, 50 μm. **d** The proportion of the wet weight of the tibialis anterior muscle in each group, n = 3 or 4. **e** Representative immunofluorescence images of FITC-CTB-labeled positive sensory neurons in the DRG; scale bar, 200 μm. **f**, **g** Percentages of FITC-CTB-labeled sensory and motor neurons in the DRG and anterior horn of the spinal cord. n = 3 or 4. All numerical data were analyzed using one-way ANOVA with Tukey’s multiple comparison tests and are represented as mean ± SEM. *P < 0.05, **P < 0.005, ***P < 0.001, ****P < 0.0001

As reinnervation of the target muscle is essential for the functional recovery of regenerated nerves, the NMJs of the extensor digitorum longus were stained with anti-NF/Syn antibodies and α-BTX to label presynapses and acetylcholine receptors (AChRs), respectively. The results showed that 37.73 ± 8.99% and 78.27 ± 5.08% of AChRs were reinnervated in the negative control group and in the positive control group, respectively. Treatment with NT-3 led to reinnervation of 78.70 ± 3.29% of AChRs, which was comparable to that of the positive control. However, the increased reinnervation of NMJs by NT-3 was blocked by c-Jun sh2, resulting in a reinnervation rate of 29.50 ± 7.41% (Fig. 4a, b). Additionally, the diameter of the anterior tibial muscle fibers in each group was investigated by H&E staining. The wet weight of the bilateral tibialis anterior muscles was measured and the wet weight ratio for each group was quantified. The results showed that the diameter of fibers and wet weight ratios of the NT-3 (5 W) and control (+) groups were similar and higher than those of the control (−) and NT-3 (5 W) + sh2 groups, consistent with that of NMJ reinnervation (Fig. 4c, d).

CTB has been used for retrograde tracing to study muscle reinnervation. Five weeks after nerve suturing, 10 µl FITC-CTB (0.4 mg/ml) was injected into the tibialis anterior muscle. One week after injection, L4 and L5 DRGs, as well as spinal cord sections, were stained for NeuN (Fig. 4e and Additional file 2: Fig. S4). The percentage of CTB-labeled neurons in the DRGs or anterior horn of the spinal cord were higher in the NT-3 (5 W) and control (+) groups. The administration of both NT-3 and sh2 resulted in reduced CTB labeling, similar to that of the negative control (Fig. 4f, g). Taken together, these results suggest that NT-3 promotes peripheral nerve regeneration after chronic denervation, mainly by maintaining a high level of c-Jun rather than NT-3 itself.

### NT-3 re-upregulates the expression of low-level c-Jun after chronic denervation and promotes peripheral nerve regeneration

**Fig. 5:**
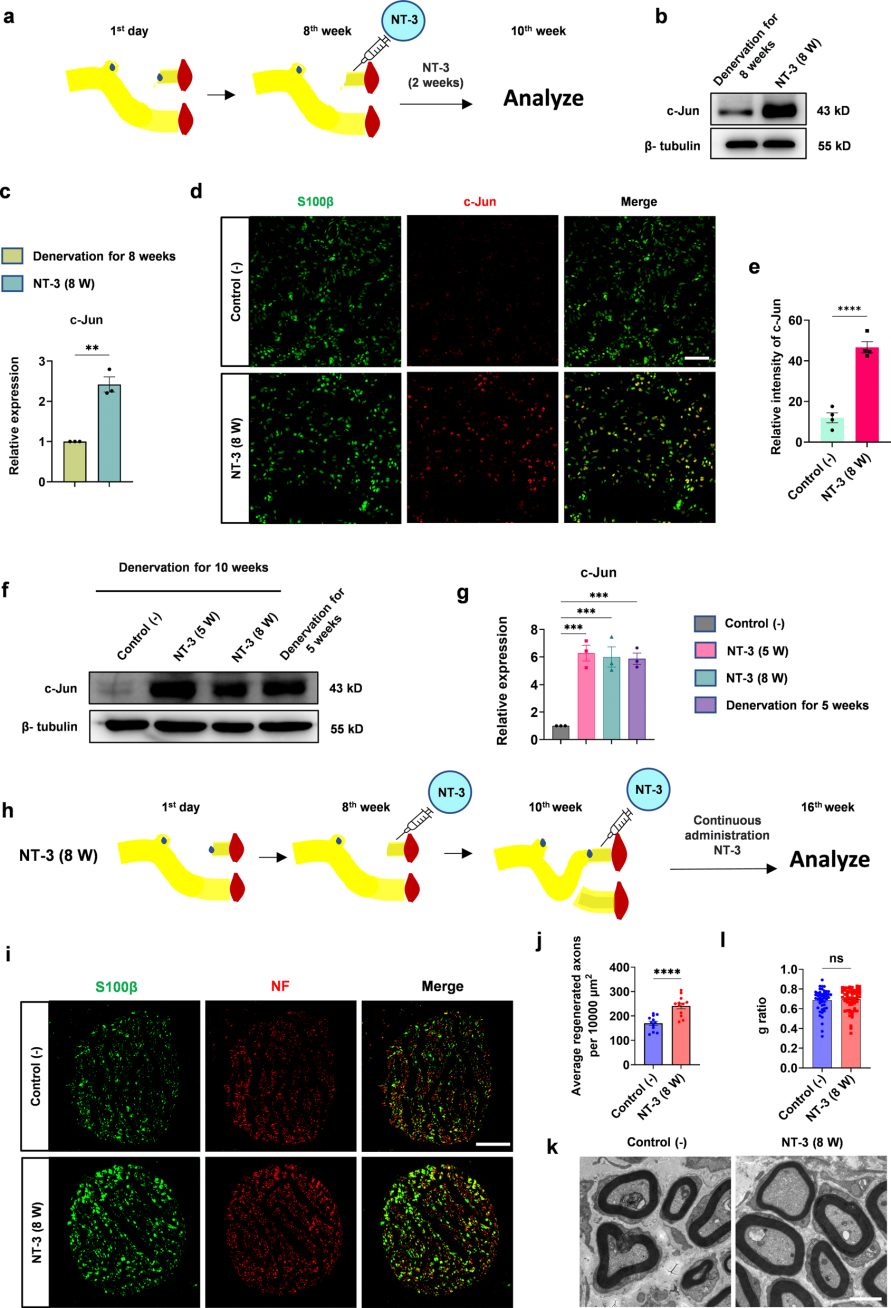
NT-3 re-upregulates the expression of low-level c-Jun and promotes peripheral nerve regeneration after chronic denervation.** a** Operational diagram of the NT-3 (8 W) group. **b** Western blotting used to analyze the expression level of c-Jun in the NT-3 (8 W) group and the distal denervated nerves for 8 weeks. **c** Relative quantitative analysis of c-Jun expression. Unpaired Student’s t-test, **P < 0.005, n = 3. **d** Immunofluorescence analysis of c-Jun expression in the distal denervated nerve; scale bar, 50 μm. **e** The expression of c-Jun analyzed by fluorescence intensity. Unpaired Student’s t-test, ****P < 0.0001, n = 4. **f** Western blotting used to analyze the expression level of c-Jun in the NT-3 (5 W), NT-3 (8 W), and control (−) groups, and the distal denervated nerves for 5 weeks. **g** Relative quantitative analysis of c-Jun expression. One-way ANOVA with Tukey’s multiple comparison test, ***P < 0.001, n = 3. **h** Schematic diagram of establishment of a regeneration model of the peripheral nerve after chronic denervation. Schematic representation of the NT-3 (8 W) group. **i** Representative images of peripheral nerve regeneration observed by immunofluorescence staining. S100β labeled myelin and NF-labeled axons; scale bar, 100 μm. **j** Statistical analysis of the number of regenerated axons per 10,000 µm^2^ in the two groups. Unpaired Student’s t-test, ****P < 0.0001, n = 12 from 4 rats. **k** Representative images of myelination of regenerated axons in each group were observed using TEM; scale bar, 2 μm. **l** Statistical analysis of myelination in the NT-3 (8 W) and control (−) groups show no significant differences. Unpaired Student’s t-test, n = 48 or 61 from 3 rats. All numerical data are presented as mean ± SEM

In daily clinical practice, the distal stump of the injured nerve is usually in a state of chronic denervation already, with c-Jun expression at a very low level. We then investigated whether NT-3 could re-upregulate the decreased c-Jun. Since c-Jun was seldom expressed in the injured distal stump at 8 weeks after denervation, 4 µg NT-3 (in 50 µl PBS) was administered locally every other day by intramuscular injection around the distal stump from week 8 and lasted for 2 weeks (the NT-3 (8 W) group) (Fig. 5a). Western blotting showed that, compared with c-Jun levels at the 8th week after denervation, the expression of c-Jun was upregulated after NT-3 treatment (Fig. 5b, c). Ten weeks after denervation, quantification of the immunofluorescence intensity also showed that c-Jun in the distal stumps was higher in the NT-3 (8 W) group than in the control (−) (Fig. 5d, e), almost comparable to that in the 5th week after denervation, when c-Jun was still abundant in the distal stump. No difference in c-Jun expression was observed between NT-3 administered at 8th week and 5th week (Fig. 5f, g), indicating that NT-3 upregulated the expression of c-Jun to a high level in distal denervated nerves.

To further determine whether upregulation of c-Jun by NT-3 could promote regeneration after chronic denervation, a peripheral nerve regeneration model was established again with NT-3 injected at the 8th week and lasting for 8 weeks (Fig. 5h). PBS was administered to the control (−) group (Additional file 2: Fig. S1b). The regenerated nerves were evaluated 1 cm distal to the suture site by immunofluorescence staining. Consistently, the number of average regenerated axons per unit area (10,000 µm^2^) in the NT-3 (8 W) group was higher than that in the control (−) group (Fig. 5i, j). Myelination of the distal nerve was assessed by TEM, which showed similar remyelination between these two groups (Fig. 5k, l).

**Fig. 6:**
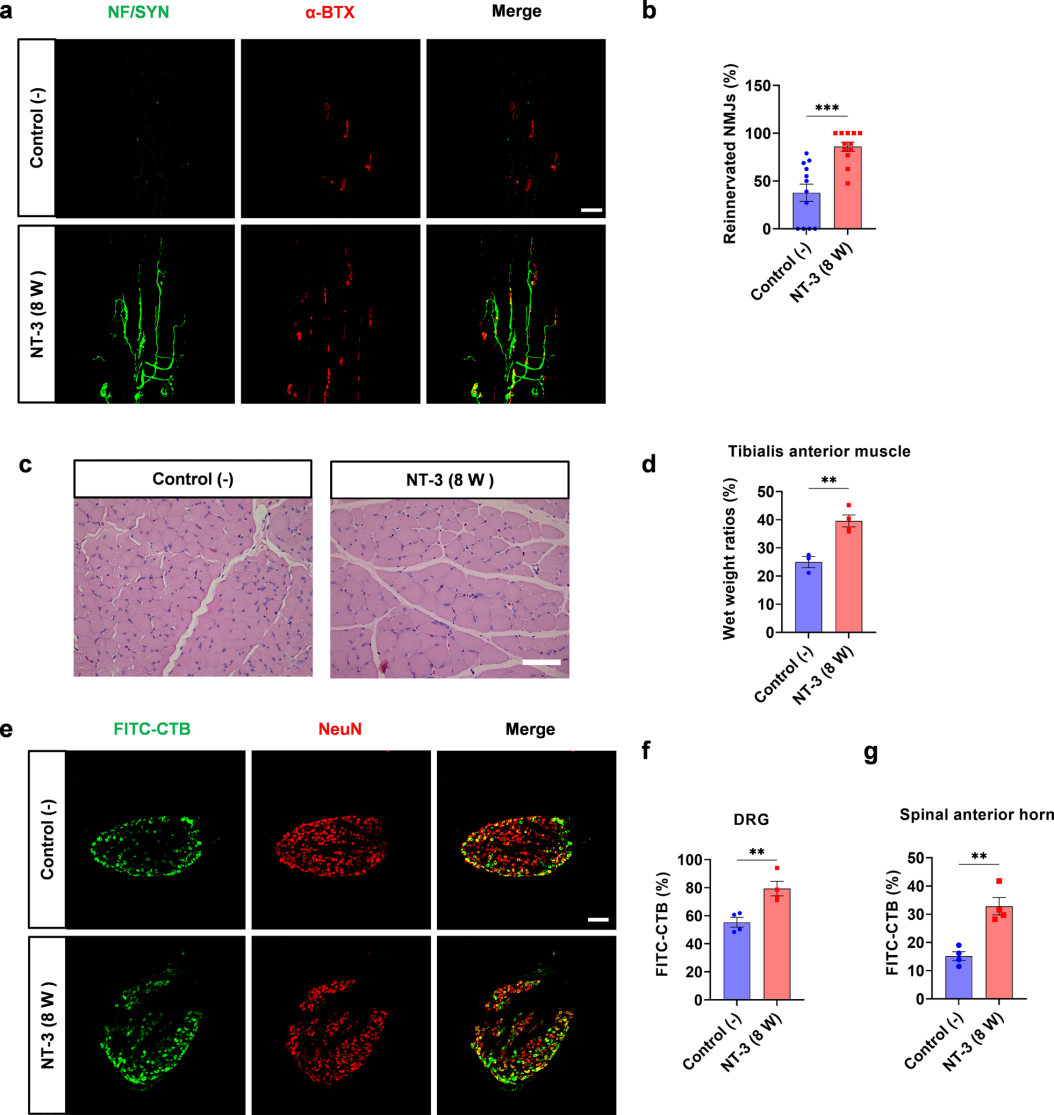
Reinnervation of target muscles by regenerated axons.** a** Representative images of NMJs using immunofluorescence staining. Green: Presynaptic NMJs and regenerated axons. Red: AChRs in the post-synapses of NMJs; scale bar, 100 μm. **b** Percentage of reinnervated NMJs per group, n = 12 from 3 rats. **c** The diameter of the anterior tibial muscle fibers in each group was shown by H&E staining, scale bar, 50 μm. **d** The proportion of wet weight of the tibialis anterior muscle in each group, n = 3 or 4. **e** Representative immunofluorescence images of FITC-CTB-labeled positive sensory neurons in DRG; scale bar, 200 μm. **f**, **g** Percentages of FITC-CTB-labeled sensory and motor neurons in the DRG and anterior horn of the spinal cord. n = 3 or 4. All numerical data were analyzed using unpaired Student’s t-test and are presented as mean ± SEM. **P < 0.005, ***P < 0.001

For reinnervation of the target muscle, NMJ staining showed that more AChRs were innervated by regenerated nerves in the NT-3 (8 W) group, with a higher percentage of NMJ reinnervation (Fig. 6a, b). Additionally, the diameter of fibers and wet weight ratio of the NT-3 (8 W) group were higher than that of the control (−) group (Fig. 6c, d). FITC-CTB injection was used to check whether the regenerated nerves formed synapses with the target muscles. Immunofluorescence staining showed higher percentages of CTB-labeled neurons in the DRG and the anterior horn of the spinal cord in the NT-3 (8 W) group (Fig. 6e–g and Additional file 2: Fig. S4). These results suggest that NT-3 could re-upregulate the reduced expression of c-Jun after chronic denervation and promote peripheral nerve regeneration.

### NT-3 upregulates c-Jun by activating the ERK pathway in Schwann cells

**Fig. 7:**
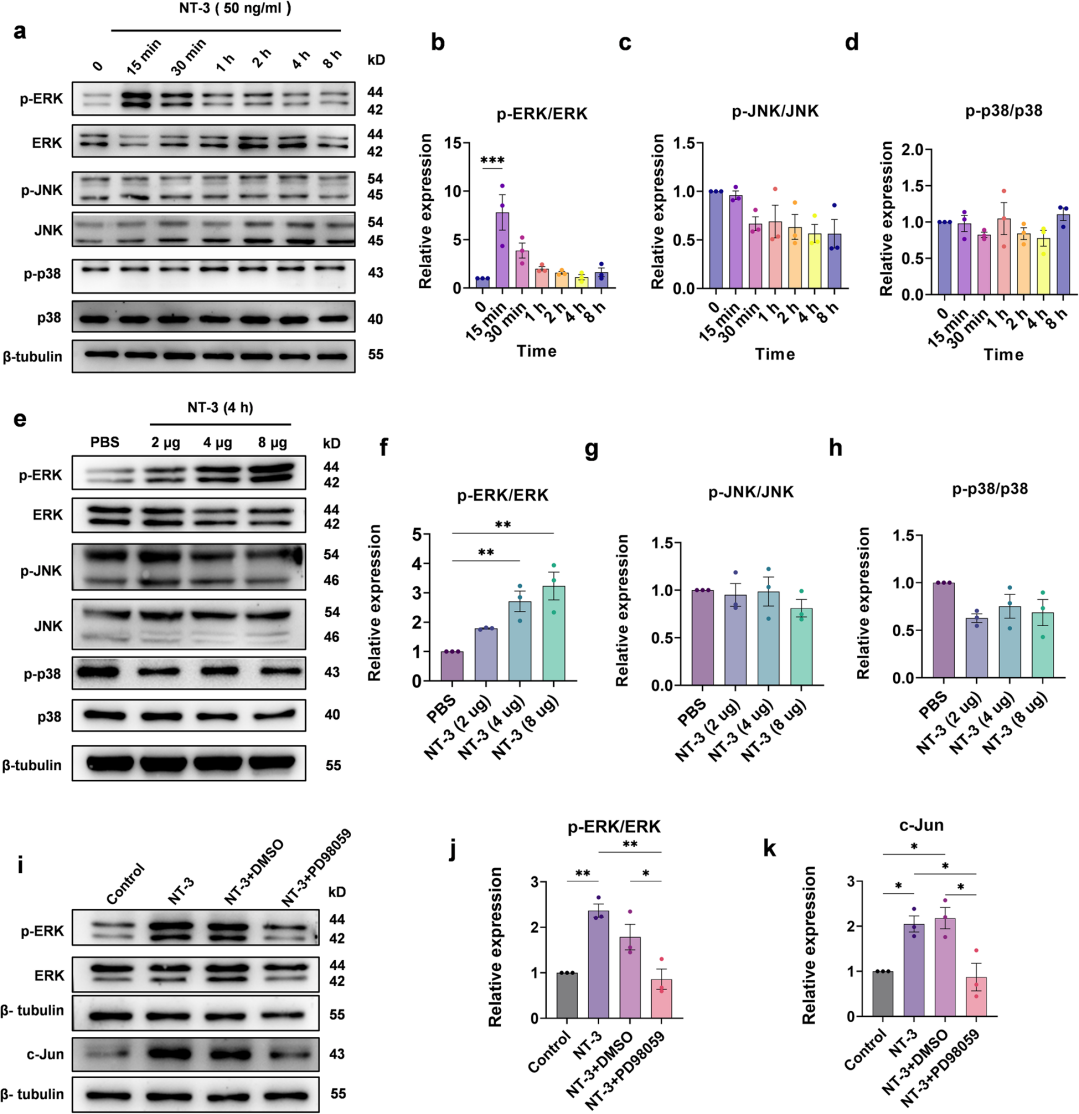
NT-3 upregulates c-Jun through the ERK pathway in Schwann cells.** a** The expression of ERK, p38, JNK, and their phosphorylation at different time points after the administration of NT-3 analyzed using western blotting. **b**–**d** Relative quantitative analysis of p-ERK/ERK, p-JNK/JNK, and p-p38/p38 show that NT-3 can only rapidly phosphorylate ERK. Data were normalized to 0 (control group). One-way ANOVA with Dunnett’s multiple comparisons test, ***P < 0.001, n = 3. **e** After common peroneal nerve transection, the distal nerve stump of the injury was denervated for 5 weeks and different doses of NT-3 were administered. After 4 h, the expression of ERK, JNK, and p38 and their phosphorylation were analyzed using western blotting. **f**–**h** Relative quantitative analysis of the expression of p-ERK/ERK, p-JNK/JNK, and p-p38/p38, Data normalized to the PBS group. One-way ANOVA with Dunnett’s multiple comparisons test, **P < 0.005, n = 3. **i** Western blot analysis of ERK, p-ERK, and c-Jun expression in the control, NT-3, NT-3 + DMSO, and NT-3 + PD98059 groups. **j** and **k** Relative quantitative analysis of p-ERK/ERK and c-Jun expression show that NT-3 can phosphorylate ERK and upregulate c-Jun, and PD98059 prevents the phosphorylation of ERK and upregulation of c-Jun caused by NT-3. The data were normalized to the control. One-way ANOVA with Tukey’s multiple comparison test, *P < 0.05, **P < 0.005, n = 3. All numerical data are presented as mean ± SEM

It has been reported that c-Jun can be upregulated by p38 MAPKs, ERK, and JNK pathways in Schwann cells [15–19]. In this study, we investigated whether NT-3 upregulates c-Jun through the activation of any of these pathways. To answer this question, NT-3 (50 ng/ml) was administered to mimic denervated Schwann cells (eighth passage) and the expression of ERK, p38, JNK and their phosphorylation were detected at 0 min, 15 min, 30 min, 1 h, 2 h, 4 h, and 8 h later (Fig. 7a). Quantification of the relative expression of p-ERK/ERK, p-p38/p38, and p-JNK/JNK (Fig. 7b–d) showed that p-ERK/ERK was significantly upregulated with the highest expression at 15 min, which was approximately eight times higher than that of the control (Fig. 7b). However, there were no differences in the expression of p-JNK/JNK and p-p38/p38 after NT-3 administration (Fig. 7c, d), suggesting that NT-3 upregulates c-Jun via the ERK pathway.

Next, NT-3 at a total amount of 2, 4, and 8 µg in 50 µl PBS was injected at the distal denervated stump on the 5th week. Four hours later, the distal stumps were subjected to western blotting to determine the expression of p-ERK/ERK, p-p38/p38, and p-JNK/JNK. Consistently, p-ERK/ERK expression increased in a dose-dependent manner after NT-3 application, and 4 µg NT-3 led to a 2.7-fold increase over the control group (Fig. 7e, f). Again, no elevation was observed in the relative expression of p-p38/p38 and p-JNK/JNK (Fig. 7g, h). These results suggest that NT-3 could significantly activate ERK both in vivo and in vitro.

Then, to determine whether NT-3 upregulates c-Jun through the ERK pathway, PD98059, an inhibitor of the ERK pathway, was used to block ERK activation. Schwann cells were pretreated with PD98059 (10 µM) or DMSO for 1 h and were then treated with NT-3 (50 ng/ml) for 15 min or 2 h as NT-3 significantly phosphorylates ERK at 15 min and upregulates c-Jun at 2 h. Western blotting showed that p-ERK/ERK expression was increased by NT-3; however, this increase was blocked by the administration of PD98059 but not DMSO. Accordingly, c-Jun was upregulated when treated with NT-3 alone. However, no increase in c-Jun was observed when cells were treated with NT-3 and PD98059 simultaneously (Fig. 7i–k). Together, these results suggest that NT-3 upregulates c-Jun, mainly through the ERK pathway.

### NT-3 activates the ERK pathway through TrkC in Schwann cells in vitro

**Fig. 8:**
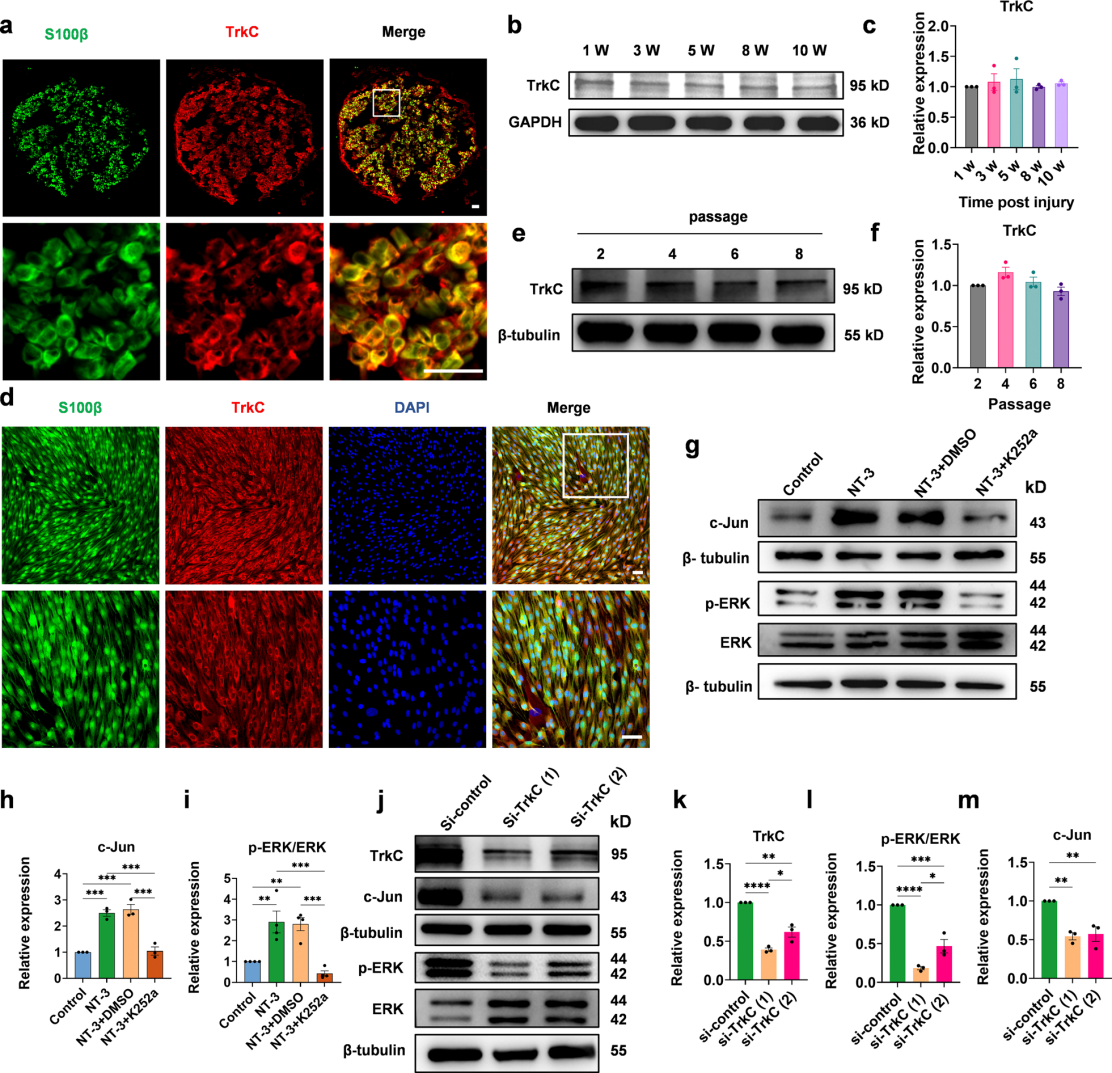
NT-3 activates ERK pathway signaling through TrkC in Schwann cells.** a** Representative images of the distal common peroneal nerve after 1 week of denervation by immunostaining with S100β and TrkC. Scale bar, 30 μm. **b** Western blotting used to detect the expression of TrkC in the injured distal stumps after 1, 3, 5, 8, and 10 weeks of denervation. **c** Relative quantitative analysis of TrkC expression. Data were normalized to 1 week. One-way ANOVA with Dunnett’s multiple comparisons test, n = 3. **d** Representative images of Schwann cells (passage 2) by immunostaining with S100β and TrkC, scale bar, 50 μm. **e** Western blot analysis of TrkC expression in Schwann cells at different passages (passages 2, 4, 6, 8). **f** Relative quantitative analysis of TrkC expression. Data were normalized to passage 2. There is no statistically significant difference. One-way ANOVA with Dunnett’s multiple comparisons test, n = 3. **g** Western blot analysis of ERK, p-ERK, and c-Jun expression in the control, NT-3, NT-3 + DMSO, and NT-3 + K252a groups. **h**, **i** Relative quantitative analysis of the expression of p-ERK/ERK and c-Jun show that K252a prevents the phosphorylation of ERK and the upregulation of c-Jun caused by NT-3. The data were normalized to the control. One-way ANOVA with Tukey’s multiple comparison test, **P < 0.005, ***P < 0.001, n = 3 or 4. **j** Western blot analysis of TrkC, ERK, p-ERK, and c-Jun expression in the si-control, si-TrkC (1), and si-TrkC (2) groups. **k**–**m** Relative quantitative analysis of the expression of TrkC, p-ERK/ERK, and c-Jun. The data were normalized to the control. One-way ANOVA with Tukey’s multiple comparison test. *P < 0.05, **P < 0.005, ***P < 0.001, ****P < 0.0001, n = 3. All numerical data are presented as mean ± SEM

TrkC has a high affinity for NT-3 and can elicit a range of responses upon binding to NT-3 [35, 36]. However, whether TrkC is present in denervated Schwann cells and whether it mediates the upregulation of c-Jun by NT-3 is unknown. To determine the expression of TrkC in the distal denervated nerve, the common peroneal nerve in rats was transected at 1, 3, 5, 8, and 10 weeks. The distal denervated nerves (1st week) were subjected to immunofluorescence staining with antibodies against TrkC and S100β. The results showed that TrkC was present in S100β + Schwann cells (Fig. 8a) and continuously expressed during chronic denervation (Fig. 8b, c). Additionally, TrkC was also consistently expressed in cultured Schwann cells (second passage) (Fig. 8d) and was not affected by cell passage in vitro (Fig. 8e, f).

To determine whether TrkC mediates the activation of the ERK pathway by NT-3, we first blocked TrkC with K252a, a protein kinase inhibitor. The expression of ERK, p-ERK, and c-Jun was detected after pretreatment with K252a (100 nM) or DMSO for 1 h, followed by the administration of NT-3 (50 ng/ml) for 15 min or 2 h, respectively (Fig. 8g). The results showed that K252a significantly reduced the upregulation of c-Jun and p-ERK induced by NT-3 (Fig. 8h, i). In addition, two siRNAs targeting TrkC (si-TrkC (1) and si-TrkC (2)) and a non-interfering siRNA (si-control) were constructed. After Schwann cells were transfected with siRNA for 3 days, NT-3 (50 ng/ml) was administered, and western blotting showed that both si-TrkCs resulted in high silencing efficiency (Fig. 8j, k) and inhibited the upregulation of c-Jun and p-ERK by NT-3 (Fig. 8l, m). These results suggested that NT-3 regulates the ERK/c-Jun pathway via TrkC.

## Discussion

Myelin and Remak Schwann cells transform into repair Schwann cells after nerve injury, which promote axonal regeneration by secreting neurotrophic factors and forming Büngner bands. However, if no axons grow into the bands of Büngner for an extended period, repair Schwann cells enter a chronic denervation state, which impairs the ability of repair Schwann cells to promote axon regeneration. Consequently, the denervated distal stump gradually loses the microenvironment that promotes axon regeneration, which is the main reason underlying poor functional recovery [37–39]. Based on this, this study aimed to find a way to maintain the repair ability of Schwann cells in chronic denervation and found that upregulating the expression of c-Jun by NT-3 administration showed potential.

c-Jun and STAT3 play important roles in Schwann cell plasticity and promote peripheral nerve regeneration after peripheral nerve injury [7, 40]. c-Jun plays a key role in maintaining the reparative phenotype of Schwann cells and promotes peripheral nerve regeneration [7]. Stable overexpression of c-Jun in Schwann cells promotes peripheral nerve regeneration after chronic denervation [9, 10]. However, in cases with scarce c-Jun expression in the distal nerve after denervation, it is largely unknown whether re-upregulating the expression of c-Jun can promote the regeneration of peripheral nerves. Here, we found that c-Jun initially increased and then decreased over time in the distal nerve following denervation. Maintaining high levels of c-Jun expression by NT-3 in the distal nerve promoted peripheral nerve regeneration, while inhibiting c-Jun expression significantly reduced nerve regeneration, consistent with an earlier report [7]. Furthermore, we observed that when c-Jun expression had already been downregulated in distal denervated stumps, upregulating c-Jun in Schwann cells by NT-3 could reactivate their repair ability to promote peripheral nerve regeneration. This further confirmed the critical role of c-Jun in peripheral nerve regeneration after chronic denervation.

Several drugs have been investigated for their ability to upregulate c-Jun in Schwann cells, and the results of these studies are promising. Purmorphamine, an agonist of the Sonic Hedgehog signaling pathway, upregulates c-Jun in Schwann cells [9]. In addition, Fingolimod, Interleukin-1 beta, and fibroblast growth factor 21 have also been shown to upregulate c-Jun in Schwann cells [41–43]. However, further studies are required to determine the safety and efficacy of these drugs in humans.

Nrg-1 can also upregulate c-Jun via the ErbB receptor [18, 44, 45], and Nrg-1 has demonstrated good safety in clinical trials [46]. However, ErbB receptors on Schwann cells are significantly reduced after chronic denervation [44, 47, 48], which makes the ErbB receptor an unstable target for Nrg-1 to upregulate c-Jun expression in denervated Schwann cells. In addition, it is unclear whether these drugs can benefit peripheral nerve regeneration during chronic denervation.

NT-3 is a member of the neurotrophin family of growth factors and plays a vital role in the development, maintenance, and survival of neurons in the nervous system. It is primarily expressed in the CNS and PNS and has been used in the treatment of various diseases [26, 29, 49–52]. However, whether NT-3 can promote peripheral nerve regeneration after chronic denervation and its underlying molecular mechanisms have not yet been studied. In this study, we found that NT-3 primarily upregulated c-Jun through the TrkC/ERK pathway to promote peripheral nerve regeneration after chronic denervation, and the upregulated c-Jun was mainly located in the nuclei of denervated Schwann cells. TrkC expression in the distal nerve did not change significantly during denervation, thus providing a stable therapeutic target for NT-3. In Schwann cells, NT-3 was found to upregulate c-Jun, mainly through the ERK pathway. Consistent with a previous report, the ERK pathway plays an important role in the plasticity of Schwann cells and peripheral nerve regeneration [19]. We also demonstrated that when the expression of c-Jun in the distal nerves was low, NT-3 could re-upregulate and maintain a high level of c-Jun.

It has been previously reported that inhibiting the proliferation of Schwann cells in the distal stump of injured peripheral nerve did not affect axonal regeneration and myelination [53, 54]. Additionally, a recent study found that aging mice exhibited a comparable number of Schwann cells in the injured distal peripheral nerve when compared to young mice, whereas axonal regeneration was compromised in aging mice with low c-Jun expression [9]. Together, these results suggested that the promotion of peripheral nerve regeneration by NT-3 might not be primarily due to the Schwann cell number but the upregulation of c-Jun to maintain the repair phenotype of Schwann cells. As mentioned previously, c-Jun could act as a negative regulator of myelination [10, 12, 13]. However, myelination of regenerated axons was similar among the groups. A possible reason might be that when the denervated Schwann cells were contacted by regenerated axons, they retransform into myelinating Schwann cells and Remak Schwann cells, where c-Jun was reduced, and NT-3 could no longer upregulate the expression of c-Jun in the retransformed Schwann cells.

NT-3 has been used in clinical phase I and II trials and has shown good safety and tolerability [27–29]. NT-3 exhibited the potential to improve the symptoms of functional constipation in patients [28]. Additionally, in individuals with Charcot-Marie-Tooth type 1 A, NT-3 has shown promise in promoting peripheral nerve regeneration [29]. These findings have paved the way for the clinical application of NT-3 in the treatment of peripheral nerve injury after chronic denervation. However, the dosing regimen of NT-3 in our study was every other day which might present certain limitations for future clinical treatment. In future studies, efforts should be made to determine a method for long-term local release of drugs around the denervated nerves.

## Conclusions

**Fig. 9:**
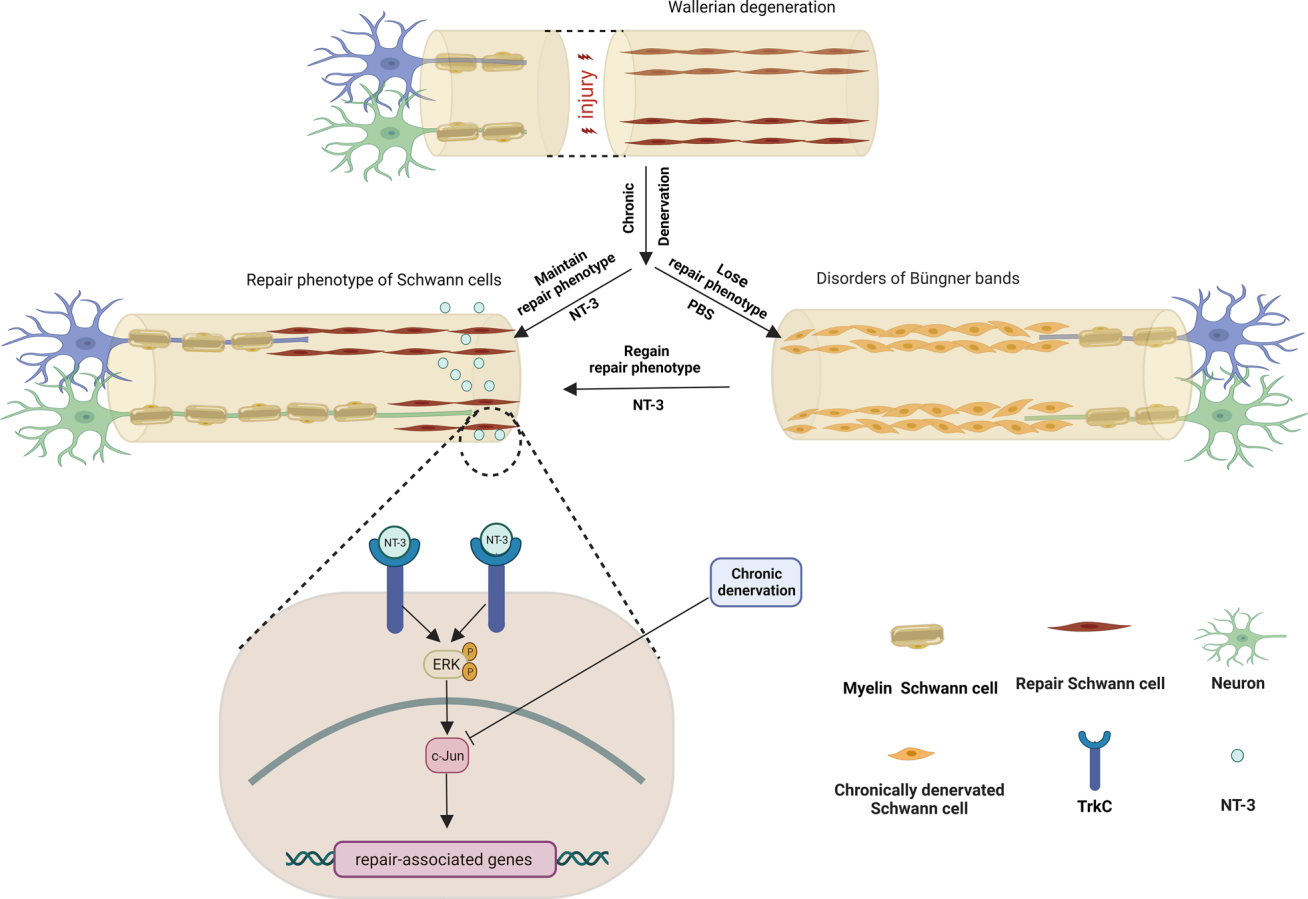
Schematic diagram of NT-3 promoting peripheral nerve regeneration after chronic denervation and related molecular mechanisms

In summary, this study characterized the variation of c-Jun expression during chronic denervation and found that NT-3 promoted peripheral nerve regeneration after chronic denervation. These effects were primarily exerted by upregulating or maintaining a high level of c-Jun rather than NT-3 itself. Mechanism studies suggested that NT-3 promoted the high expression of c-Jun via the TrkC/ERK pathway in Schwann cells. TrkC was consistently expressed in the distal nerve during denervation, making it a potential therapeutic target for NT-3 (Fig. 9). These findings provide a potential therapeutic target for the clinical treatment of peripheral nerve injury after chronic denervation.

### Supplementary Information

**Additional file 1: Table S1.** Details of the sources and applications of the antibodies.

**Additional file 2: Figure S1.** Establishment of regeneration model of peripheral nerve after chronic denervation. (a) Schematic diagram of a regeneration model of peripheral nerves after chronic denervation. (b) Schematic diagram of specific operations for each group. The purpose of this study was to explore whether NT-3 could promote peripheral nerve regeneration by maintaining high levels of c-Jun. Four groups were designed: negative control group (control (−)), NT-3 (5 W) group, NT-3 (5 W) + sh2, and positive control group (control (+)). **Figure S2.** Designing shRNA sequences and constructing AAV vectors. Sequences to target the expression of c-Jun, and a non-interfering control shRNA were designed. The constructed shRNAs were inserted into the selected interference vector (pAAV-U6-shRNA/spgRNA v2.0-CMV-EGFP-WPRE). A control vector expressing a non-targeting shRNA (pAAV-U6-ctrl. sh-CMV-eGFP) was used as a control. **Figure S3.** The viability of Schwann cell was measured assay by CCK-8 kit at different timepoints. The CCK-8 assay was used to assess the effects of NT-3 ((50 ng/ml) on Schwann cell viability at 8, 16, 24 and 48 h. Two-way ANOVA with Sidak’s multiple comparisons test; **P < 0.005. n = 4. **Figure S4.** FITC-CTB-labeled motor neurons in the anterior horn of the spinal cord. Representative immunofluorescence image of FITC-CTB-labeled motor neurons in the anterior horn of the spinal cord., Scale bar, 200 μm.

## Data Availability

The datasets used and/or analyzed during the current study are available from the corresponding author on reasonable request.

## Abbreviations

NT-3: Neurotrophin-3
TEM: Transmission electron microscopy
AAV: Adeno-associated virus
H&E: Hematoxylin and eosin
DMSO: Dimethyl sulfoxide
PBS: Phosphate buffer saline
FITC-CTB: Fluorescein isothiocyanate-conjugated cholera toxin subunit B
PFA: Paraformaldehyde
BSA: Bovine serum albumin
VG: Viral genomes
NMJ: Neuromuscular junction
NF: Neurofilament
Syn: Synapsin
PLL: Poly-l-lysine
α-BTX: α-Bungarotoxin
AChR: Acetylcholine receptor
L: Lumbar
DRG: Dorsal root ganglion
TrkC: Tropomyosin receptor kinase C
CCK-8: Cell Counting Kit-8
p38 MAPK: p38 mitogen-activated protein kinase
JNK: Jun N-terminal kinase
CNS: Central nervous system
PNS: Peripheral nervous system
siRNA: Small-interfering RNA
Nrg-1: Neuregulin-1
shRNA: Small hairpin RNA
