# Supplementary material for: Neurotrophin-3 promotes peripheral nerve regeneration by maintaining a repair state of Schwann cells after chronic denervation via the TrkC/ERK/c-Jun pathway
Source: J Transl Med. 2023 Oct 17;21:733. doi: 10.1186/s12967-023-04609-2 (PMC10583391; doi:10.1186/s12967-023-04609-2)
Supplement: Supplementary file 1 — Additional file 1: Table S1. Details of the sources and applications of the antibodies. [file 12967_2023_4609_MOESM1_ESM.docx]

**Additional file 1**

Table S1

| Antibody | Applications/Dilution | Catalog | Company |
| --- | --- | --- | --- |
| TrkC(NTRK3) | WB 1:1000  IF 1:200 | MB0351S | Abmart |
| c-Jun | WB 1:1000  IF 1:100 | T550905 | Abmart |
| ERK | WB 1:1000 | 4695 | Cell Signaling Technology |
| p-ERK | WB 1:2000 | 4370 | Cell Signaling Technology |
| JNK | WB 1:1000 | 9258 | Cell Signaling Technology |
| p-JNK | WB 1:1000 | 4668 | Cell Signaling Technology |
| p38 MAPK | WB 1:1000 | 8690 | Cell Signaling Technology |
| p-p38 MAPK | WB 1:1000 | 4511 | Cell Signaling Technology |
| GAPDH | WB 1:5000 | HC301 | TransGen Biotech |
| β-tubulin | WB 1:5000 | HC101 | TransGen Biotech |
| HRP-labeled Goat Anti-Rabbit IgG(H+L) | WB 1:1000 | A0208 | Beyotime Biotechnology |
| HRP-labeled Goat Anti-Mouse IgG(H+L) | WB 1:1000 | A0216 | Beyotime Biotechnology |
| Neurofilament (C28E10) (NF) | IF 1:300 | 2837S | Cell Signaling Technology |
| Synapsin (SYN) | IF 1:800 | 5297S | Cell Signaling Technology |
| S100β | IF 1:500 | S2532 | Sigma-Aldrich |
| S100β | IF 1:100 | ab52642 | Abcam |
| NeuN (D4G4O) | IF 1:100 | 24307S | Cell Signaling Technology |
| anti-Mouse IgG (H+L) labeled with Alexa-488 | IF 1:1000 | A32766 | Invitrogen |
| Anti-rabbit IgG (H+L) labeled with Alexa-488 | IF 1:500 | A11008 | Invitrogen |
| Anti-Mouse IgG(H+L) labeled with Alexa-546 | IF 1:500 | A10040 | Invitrogen |
| Anti-Rabbit IgG(H+L) labeled with Alexa-546 | IF 1:500 | A11035 | Invitrogen |
| anti-Mouse IgG (H+L) labeled with Alexa-647 | IF 1:500 | A-21235 | Thermo Fisher Scientific |
