# Supplementary material for: Neurotrophin-3 promotes peripheral nerve regeneration by maintaining a repair state of Schwann cells after chronic denervation via the TrkC/ERK/c-Jun pathway
Source: J Transl Med. 2023 Oct 17;21:733. doi: 10.1186/s12967-023-04609-2 (PMC10583391; doi:10.1186/s12967-023-04609-2)

Supplemental Figure 1 Establishment of regeneration model of peripheral nerve after chronic denervation

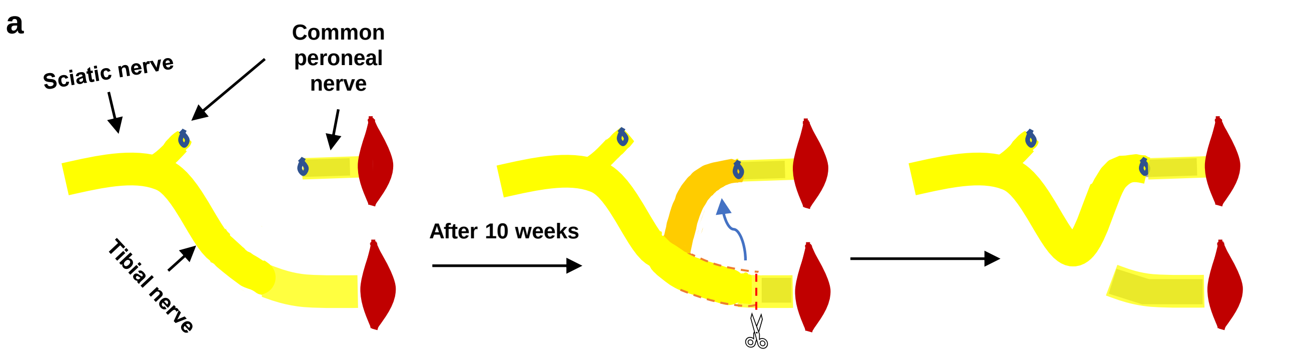

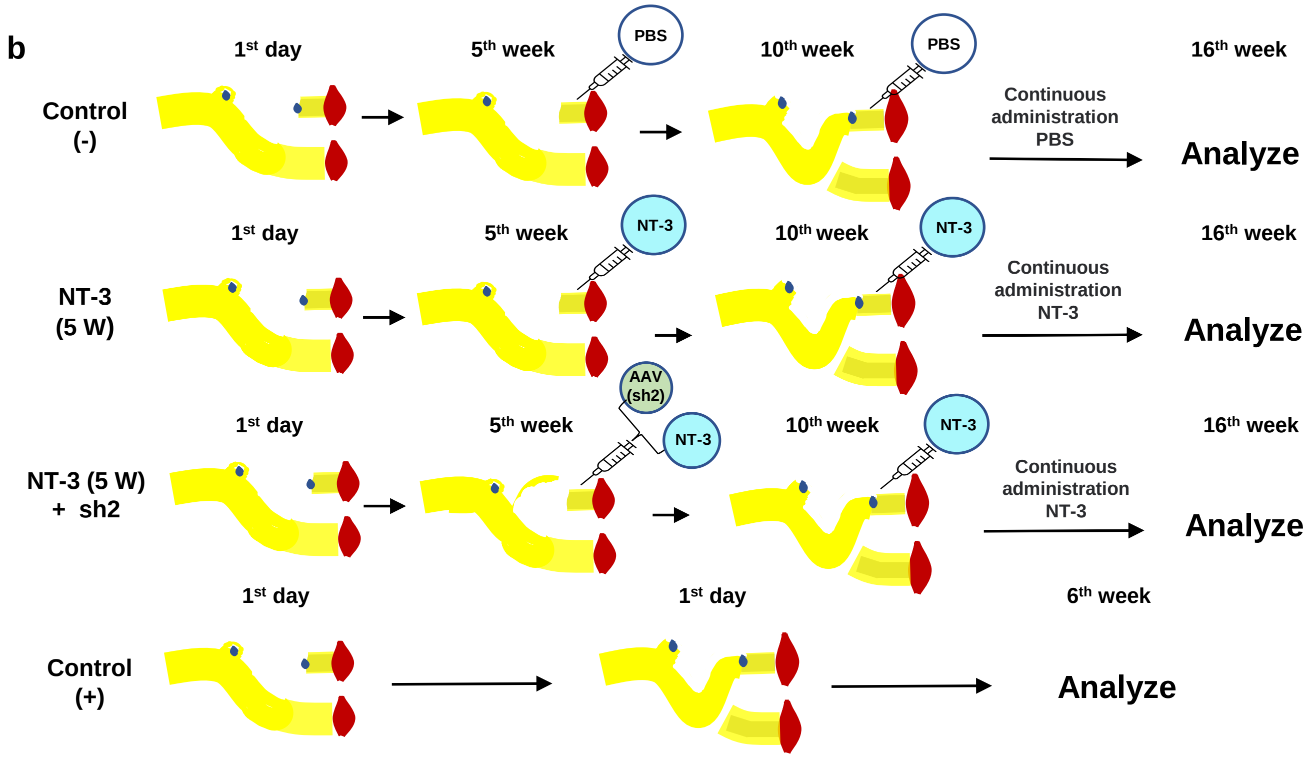

Supplemental Figure 2 Designing shRNA sequences and constructing AAV vectors.

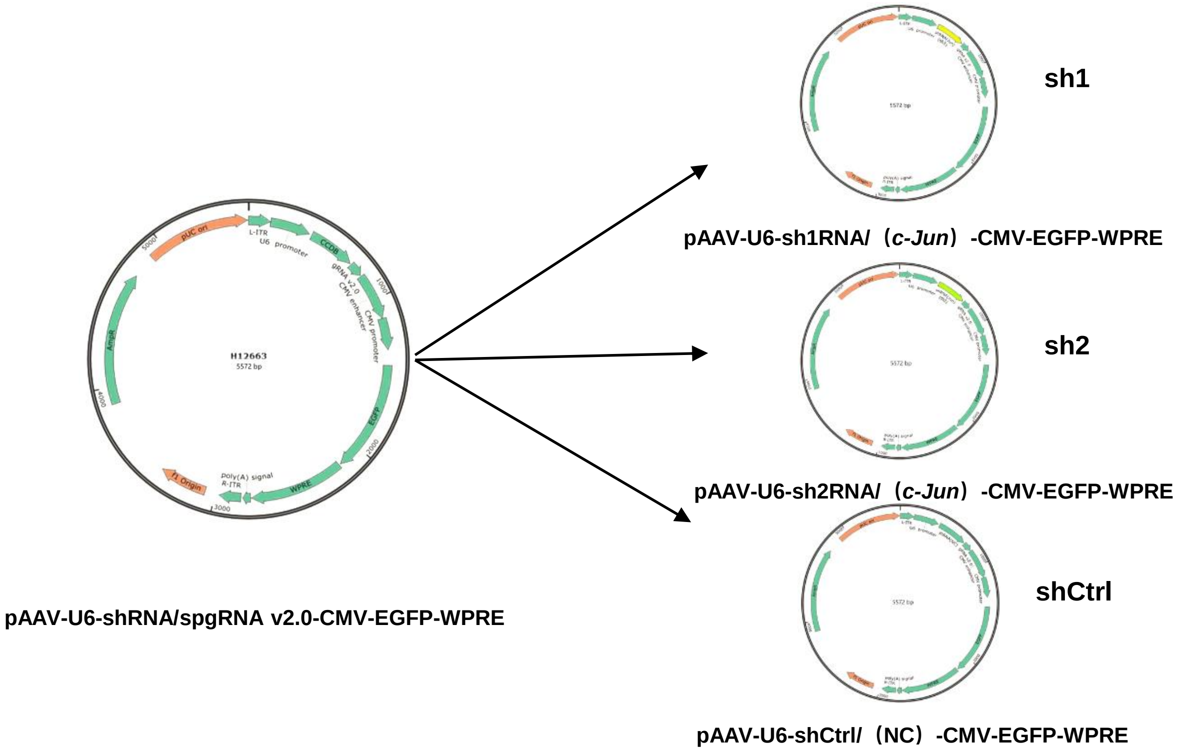

Supplemental Figure 3 The viability of Schwann cell was measured by CCK-8 kit assay at different timepoints

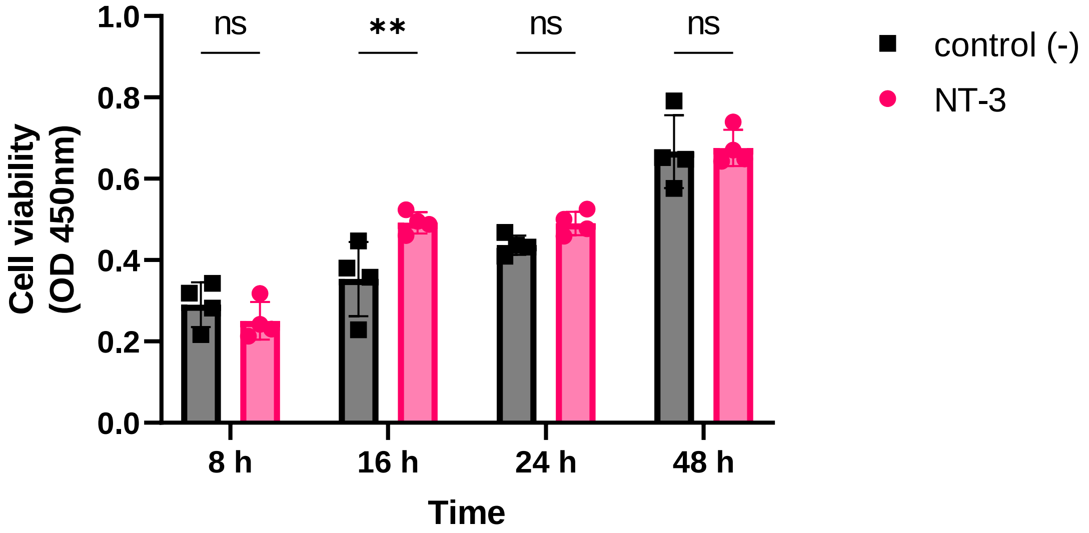

Supplemental Figure 4 FITC-CTB-labeled motor neurons in anterior horn of the spinal cord

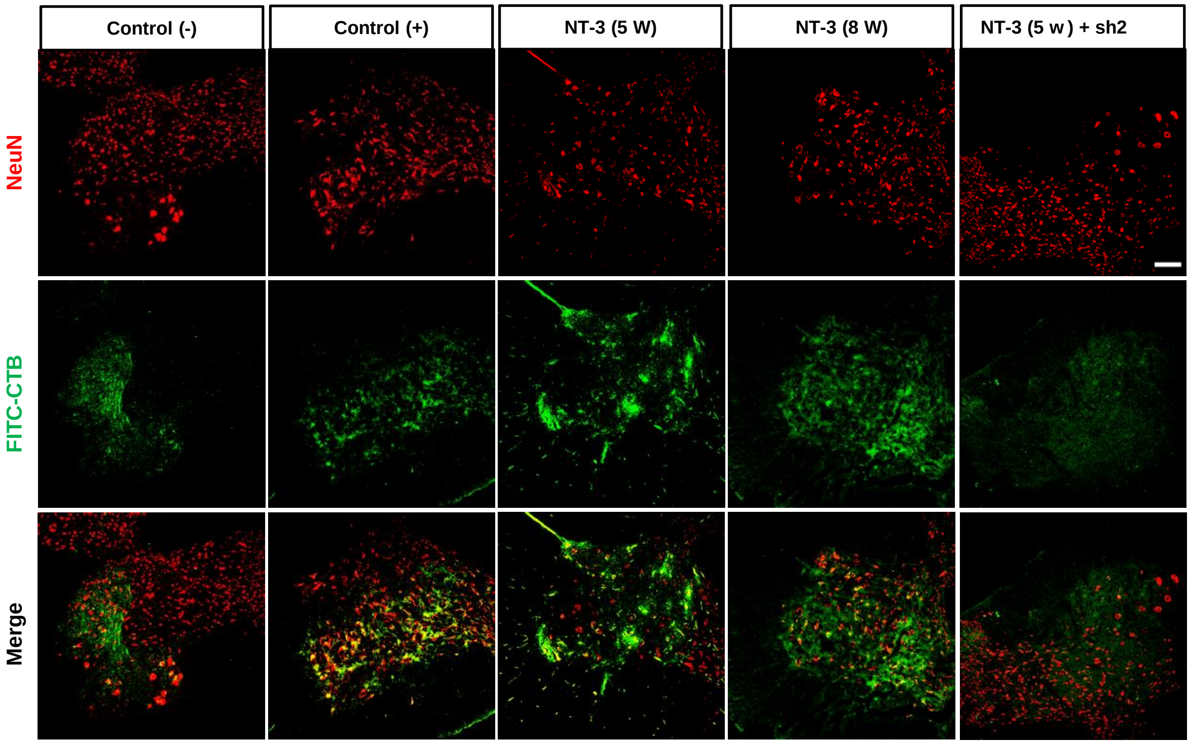

Supplement: Supplementary file 2 — Additional file 2: Figure S1. Establishment of regeneration model of peripheral nerve after chronic denervation. (a) Schematic diagram of a regeneration model of peripheral nerves after chronic denervation. (b) Schematic diagram of specific operations for each group. The purpose of this study was to explore whether NT-3 could promote peripheral nerve regeneration by maintaining high levels of c-Jun. Four groups were designed: negative control group (control (−)), NT-3 (5 W) group, NT-3 (5 W) + sh2, and positive control group (control (+)). Figure S2. Designing shRNA sequences and constructing AAV vectors. Sequences to target the expression of c-Jun, and a non-interfering control shRNA were designed. The constructed shRNAs were inserted into the selected interference vector (pAAV-U6-shRNA/spgRNA v2.0-CMV-EGFP-WPRE). A control vector expressing a non-targeting shRNA (pAAV-U6-ctrl. sh-CMV-eGFP) was used as a control. Figure S3. The viability of Schwann cell was measured assay by CCK-8 kit at different timepoints. The CCK-8 assay was used to assess the effects of NT-3 ((50 ng/ml) on Schwann cell viability at 8, 16, 24 and 48 h. Two-way ANOVA with Sidak’s multiple comparisons test; **P < 0.005. n = 4. Figure S4. FITC-CTB-labeled motor neurons in the anterior horn of the spinal cord. Representative immunofluorescence image of FITC-CTB-labeled motor neurons in the anterior horn of the spinal cord., Scale bar, 200 μm. [file 12967_2023_4609_MOESM2_ESM.pdf]
